# Characterizing the effects of Dechlorane Plus on β-cells: a comparative study across models and species

**DOI:** 10.1080/19382014.2024.2361996

**Published:** 2024-06-04

**Authors:** Kyle A. van Allen, Noa Gang, Myriam P. Hoyeck, Ineli Perera, Dahai Zhang, Ella Atlas, Francis C. Lynn, Jennifer E Bruin

**Affiliations:** a Department of Biology & Institute of Biochemistry, Carleton University, Ottawa, Ontario, Canada; b Diabetes Research Group, BC Children’s Hospital Research Institute, Vancouver, BC, Canada; c Environmental Health Science and Research Bureau, Health Canada, Ottawa, ON, Canada

**Keywords:** Dechlorane Plus, diabetes, islets, persistent organic pollutants, stem cells, β-cells

## Abstract

Epidemiological studies consistently link environmental toxicant exposure with increased Type 2 diabetes risk. Our study investigated the diabetogenic effects of a widely used flame retardant, Dechlorane Plus (DP), on pancreatic β-cells using rodent and human model systems. We first examined pancreas tissues from male mice exposed daily to oral gavage of either vehicle (corn oil) or DP (10, 100, or 1000 μg/kg per day) and fed chow or high fat diet for 28-days *in vivo*. DP exposure did not affect islet size or endocrine cell composition in either diet group. Next, we assessed the effect of 48-hour exposure to vehicle (DMSO) or DP (1, 10, or 100 nM) *in vitro* using immortalized rat β-cells (INS-1 832/3), primary mouse and human islets, and human stem-cell derived islet-like cells (SC-islets). In INS-1 832/3 cells, DP did not impact glucose-stimulated insulin secretion (GSIS) but significantly decreased intracellular insulin content. DP had no effect on GSIS in mouse islets or SC-islets but had variable effects on GSIS in human islets depending on the donor. DP alone did not affect insulin content in mouse islets, human islets, or SC-islets, but mouse islets co-exposed to DP and glucolipotoxic (GLT) stress conditions (28.7 mM glucose + 0.5 mM palmitate) had reduced insulin content compared to control conditions. Co-exposure of mouse islets to DP + GLT amplified the upregulation of *Slc30a8* compared to GLT alone. Our study highlights the importance and challenges of using different *in vitro* models for studying chemical toxicity.

## 1. Introduction

The International Diabetes Federation predicts that global prevalence of adults living with Type 2 diabetes (T2D) will increase from approximately 536 million to 783 million by 2045.^1^ T2D is a metabolic disorder characterized by hyperglycemia due to insufficient insulin secretion from pancreatic β-cells and/or peripheral insulin resistance. While lifestyle factors and genetic predisposition both contribute to T2D risk, they fall short of explaining the dramatic surge in T2D cases worldwide^2^. Mounting epidemiological studies report associations between exposure to environmental contaminants and increased T2D incidence.^3–7^

Persistent organic pollutants (POPs) are human-made chemicals characterized by long-half lives, resistance to degradation, and the ability to bio-magnify across trophic levels.^8^ Brominated flame retardants were widely used for decades until being classified as POPs by the Stockholm Convention and flagged for their endocrine disrupting properties.^9,10^ Many brominated flame retardants were subsequently replaced by chlorinated flame retardants, such as Dechlorane Plus (DP), which is used in textiles, electronic wiring, and plastics.^11^ However, the Stockholm Convention recently added DP to their list of POPs and recommended that regulatory interventions be implemented to prevent further DP contamination.^12^ For now, the manufacturing and use of DP in North America remains unregulated. DP is detected in the dust of residential homes in Ottawa, Canada at concentrations of 2.3–182 ng/g.^13^ DP has also been detected in human breast milk (0.98 ng/g, lipid), hair (4.08–2159 ng/g, dry weight) and serum (1.2 ng/g − 25.4 ng/g, lipid) of Canadian adults.^13–19^ Unfortunately, little is known about the effects of DP on human health, which hampers informed regulatory decision-making.

While there are no published epidemiological studies investigating the relationship between DP exposure and metabolic health outcomes, exposure to brominated flame retardants has been associated with increased T2D incidence.^6,20^ Additionally, experimental studies have demonstrated that brominated flame retardants act as endocrine disruptors *in vivo*^21–23^ and *in vitro*.^9,10,24^ Chronic low-dose exposure to various brominated flame retardants led to fasting hyperglycemia and altered plasma insulin levels in rodents.^21–23^ Additionally, treatment of an immortalized β-cell line (INS-1E) with two brominated flame retardants, PBDE-47 and PBDE-85, increased insulin secretion *in vitro*.^24^ To our knowledge, only one published study has investigated the effects of DP on glucose homeostasis; Peshdary *et al*. reported that 28-day exposure of male mice to daily low-dose DP increased random-fed plasma insulin levels in chow-fed mice and exacerbated glucose intolerance in high-fat diet (HFD)-fed mice.^25^ These data suggest that much like brominated flame retardants, DP may also act as an endocrine disruptor, possibly via dysregulated insulin secretion. However, endocrine pancreas morphology and β-cell function were not assessed by Peshdary *et al*.^25^

In the present study, we investigated the effect of DP on pancreatic β-cells *in vivo* and *in vitro*. We first characterized islet morphology and endocrine cell composition in pancreas tissues from male mice exposed to vehicle or DP and chow or HFD *in vivo*^25^. Next, we characterized the direct effects of DP on glucose-stimulated insulin secretion (GSIS), insulin content, and gene expression *in vitro* using diverse cell models, including an immortalized rat β-cell line (INS-1 832/3 cells), primary islets isolated from mice and human organ donors, and human stem cell-derived islet-like cell clusters (SC-islets). To further investigate the HFD-dependent effects reported by Peshdary *et al*., we also explored the effects of DP on mouse islets cultured under glucolipotoxic (GLT) stress conditions. Our studies provide important insight into the advantages and limitations of different β-cell models for toxicology assessments.

## 2. Materials and methods

### 2.1. Immunofluorescence staining and quantification of mouse pancreas tissues

Paraffin-embedded sections of mouse pancreas tissues were kindly provided by Dr. Ella Atlas (Health Canada). In brief, 8-week-old male C57BL/6N mice were fed a standard chow diet or 60% HFD and simultaneously received daily gavage of corn oil (vehicle control) or DP (10, 100, or 1000 μg/kg per day) for 28 days, as previously described.^25^ Whole pancreas was harvested at day 28 (*n* = 3–8 mice per condition) and stored in Bouin’s fixative (Sigma, #HT10132) prior to paraffin-embedding and sectioning (5 µm). The animal study was approved by the Health Canada animal care committee protocol # 2018-006.

Immunofluorescent staining was performed as previously described using standard antigen retrieval in 10 mM citrate buffer at 95°C for 10 minutes.^26^ The following primary antibodies were used: rabbit anti-insulin (Cell Signaling, C27C9, #3014; Danvers, MA, dilution 1:200), mouse anti-glucagon (Sigma-Aldrich, #G2654, 1:250), guinea pig anti-insulin (Thermofisher Scientific, #PA1–26938; Waltham, MA, 1:100), and mouse anti-proinsulin (DSHB, #GS-9A8-S; Iowa City, Iowa, 1:50). The following secondary antibodies were used: goat anti-rabbit IgG (H+L) AF568 (Life Technologies, #A11011; Carlsbad, CA, 1:1000), goat anti-mouse IgG (H+L) AF488 (Life Technologies, #A11029, 1:1000), and goat anti-guinea IgG (H+L) AF647 (Life Technologies, #A21450, dilution 1:1000).

For islet morphology quantification, the entire pancreas section was imaged with an Axio Observer 7 microscope so that every islet within a tissue section could be quantified. Islet numbers ranged from 4 to 54 islets per mouse and the average of all islet measurements was reported for each biological replicate. Immunofluorescence area per islet was manually quantified using Zen Blue 2.6 software (Carl Zeiss, Oberkochen Germany). Immunoreactivity above background levels was used to identify hormone^+^ (insulin, proinsulin, glucagon) cells within each islet for subsequent quantification. The % hormone^+^ area was quantified as [(hormone^+^ area per islet)/(islet size) x 100].

### 2.2. Chemical preparation

Dechlorane Plus (AccuStandard, New Haven, CT, USA), kindly provided by Dr. Atlas, was solubilized in Dimethyl Sulfoxide (DMSO; Sigma-Aldrich, #2768455-1 L, St Louis, MO) to a final concentration of 100 µM. The solution was kept at 4°C in amber vials for long-term storage. Final exposure concentrations of 1, 10, and 100 nM DP were chosen based on physiologically relevant concentrations found in human breast milk, serum and hair.^14,18,19^

### 2.3. Cell culture and treatment

#### 2.3.1. INS-1 832/3 cells

INS-1 832/3 cells (generously provided by Dr. Mathieu Ferron, Institut de recherches cliniques de Montréal)^27^, an immortalized pancreatic insulinoma β-cell line, were cultured in RPMI 1640 supplemented with L-glutamine (Corning, #MT10040CV, Tewsbury, MA), 10% heat-inactivated fetal bovine serum (FBS; Sigma-Aldrich #F1051-500 ML), 50 µM 2-mercaptoethanol (Sigma-Aldrich, #M3148-100 ML), 10 mM HEPES (Fisher, #BP310–500), and 1 mM sodium pyruvate (Sigma-Aldrich, #S8636-100 ML). Cells were maintained at 37°C and 5% CO_2_. Cells were tested and confirmed to be free of mycoplasma contamination using the MycoAlert Mycoplasma Detection kit (Lonza, #CA11006554; Basel, Switzerland), as per the manufacturer's instructions.

For all experiments, INS-1 832/3 cells (Passage = 6–11) were seeded into a 96-well plate at 3 × 10^4^ cells/well and allowed to attain 70% confluency before starting treatment. Cells were exposed to vehicle (DMSO) or DP (1, 10, or 100 nM) for 48 hours. At 48 hours, treatment media was removed and cells were washed 3 times with phosphate buffered saline (PBS; Sigma Aldrich, #D8662) prior to performing a static GSIS assay to assess β-cell function, as described in 2.4.

#### 2.3.2. Mouse islets

Islets were obtained from 11–19-week-old C57BL/6 male mice (colony bred at Carleton University) with either 6J genotype (Figure 5(a-d)) or mixed 6J and 6N genotype (Figure 5(e-j), 6). All experiments involving primary mouse islets were approved by the Carleton University Animal Care Committee (AUP #113152 and #114477) and carried out in accordance with the Canadian Council on Animal Care guidelines. Islets were isolated by pancreatic duct injection with collagenase (1,000 units/ml; Sigma Aldrich, #C7657) dissolved in Hanks’ balanced salt solution (HBSS: 137 mM NaCl, 5.4 mM KCl, 4.2 mM NaH_2_PO_4_, 4.1 mM KH_2_PO_4_, 10 mM HEPES, 1 mM MgCl_2_, 5 mM dextrose, pH 7.2), as previously described^28^. Briefly, pancreas tissues were incubated at 37°C for 10.5–11 minutes, agitated, and the collagenase reaction quenched with ice-cold HBSS with 1 mM CaCl_2_. Pancreas tissues were pelleted, then washed with HBSS +1 mM CaCl_2_ and resuspended in complete media [RPMI 1640 (Corning, #11875–0093) supplemented with 10% FBS (Sigma-Aldrich, #F1051) and 1% penicillin-streptomycin (Corning #30–002-CI)]. Islets were isolated using a Histopaque gradient (Sigma-Aldrich, #10771), filtered with a Progene 70 µm cell strainer (Ultident Scientific, #71–229483-ULT, Montreal, QC), and then handpicked under a dissecting scope to a purity >95%. Isolated islets were cultured overnight at 37°C and 5% CO_2_ in fresh complete RPMI media to allow for recovery.

Glucolipotoxicity (GLT) conditions were generated with 28.7 mM glucose + 0.5 mM palmitate in complete RPMI media. A 20 mM palmitate stock solution was generated by first solubilizing palmitate (Sigma, #P5585) in 33 mM NaOH. Solubilized 20 mM palmitate was then complexed with bovine serum albumin (BSA) (Sigma, #A7030) at 2:1 ratio of palmitate:BSA to generate a 5 mM palmitate working stock solution. The complexed palmitate:BSA solution was then added to complete RPMI media supplemented with D-(+)-glucose solution (Sigma, #G8769).

A subset of mouse islets were exposed to vehicle (DMSO) or 1 nM DP for 48-hours and dynamic insulin secretion was assessed using perifusion, as described in Section 2.5. Another subset of mouse islets were cultured in media containing either DMSO, 1 nM DP alone, GLT alone, or GLT + 1 nM DP. These islets were assessed by perifusion as in Section 2.5, lysed to measure insulin content as in Section 2.4, or stored for qPCR as in Section 2.6.

#### 2.3.3. Human donor islets

Human donor islets were isolated at the Alberta Diabetes Institute IsletCore (www.isletcore.ca) and shipped overnight to Ottawa in CMRL media (Fisher Scientific, #11-530-037) supplemented with 30% v/v BSA (Equitech Bio Inc., #BAL62; Kerrville, TX), 1X Insulin-Transferrin-Selenium (Corning, #25800CR), 1X GlutaMAX (ThermoFisher, #35050061), and 0.5% penicillin-streptomycin (Lonza, #09-757F). Upon arrival, islets were transferred to low glucose DMEM media (LG-DMEM; Fisher Scientific, #11-885-084) supplemented with 10% FBS (Sigma-Aldrich #F1051-500 ML) and 1% penicillin-streptomycin (Gibco, #15140122; Billings, MT) and cultured overnight to allow for recovery. All research using human islets was approved by the Research Ethics Board at Carleton University. See Supplemental Table S1 for donor characteristics.

Human islets were hand-picked into LG-DMEM media containing vehicle (DMSO) or DP (1 or 10 nM). Islets were treated for 48 hours, and islet function was subsequently assessed by static GSIS, as in Section 2.4.

#### 2.3.4. Human stem cell-derived islet-like cells

SC-islets were derived from INS-2A-EGFP WA01 human embryonic stem cells by Dr. Francis Lynn (University of British Columbia, BC, Canada) and shipped overnight to Ottawa. Briefly, INS-2A-EGFP cells were differentiated for 27 days according to a protocol adapted from Balboa *et al*.^29–31^ The final cell product is a mixture of pancreatic endocrine cells, including ~70% INS-GFP^+^ cells. The fully differentiated SC-islet cells used in these experiments ranged from day 37–55 of the differentiation protocol.

After SC-islets arrived at Carleton University, cells were maintained in “Stage 6” media [CMRL-1066 (VWR, # CA45001–114; Radnor, PA), 20 g/L BSA (Sigma-Aldrich, #10775835001), 1X GlutaMAX (Gibco, #35050079), 1% penicillin-streptomycin (Gibco, #15140122), 0.5 mM pyruvate (Sigma-Aldrich, #S8636–100 mL), 0.5X Insulin Transferrin-Selenium-X (Thermo Fisher, #51500056), 35 nM zinc sulfate (Sigma-Aldrich, #Z0251-100 G), 1 mM N-acetyl-L-cysteine (Sigma-Aldrich, #A9165-5 G), 10 ug/L heparin (Sigma-Aldrich, #H3149-10KU), 1:2000 Trace elements A (Corning, 25–021-CI), 1:20,000 Trace Elements B (Corning 25–022-CI, #MT99175CI), 10 nM T3 (Sigma, #T6397-100 MG), 0.5 µM ZM447439 (Cedarlane Labs, #S1103-10 MM/1 ML; Burlington, ON), and 1:2000 lipid concentrate (Fisher Scientific, #11905031)]. SC-islets were maintained at 37°C and 5% CO_2_ on a rocker (106 RPM) until analyses, with media changes every 2 days.

SC-islets were hand-picked and transferred to 6-well non-TC treated plates (VWR, #10861–554) for treatment with either vehicle (DMSO) or DP (1, 10, or 100 nM) for 48 hours. Clusters were maintained on a rocker (106 RPM) throughout the treatment. Following the 48 hour treatment, SC-islets were used to assess insulin secretion following static incubations in low glucose, high glucose, and KCl (as described in section 2.4), and total insulin content in cell lysates. Separate differentiation batches were considered “biological replicates.”

### 2.4. Static insulin secretion assays

We conducted a static GSIS assay on INS-1 832/3 cells, human donor islets, and SC-islets following exposure to DMSO or DP. For adherent INS-1 832/3 cells, the GSIS assay was conducted in a 96-well culture plate (*n* = 6 technical replicates per biological replicate, *n* = 4 biological replicates). For human islets, 25 islets per replicate were transferred to a 1.5 mL microcentrifuge tube (*n* = 5–6 technical replicates per condition for each donor). Cells were first washed with pre-warmed Krebs-Ringer bicarbonate buffer (KRBB) containing 0 mM glucose (115 mM NaCl, 5 mM KCl, 24 mM NaHCO_3_, 2.5 mM CaCl_2_, 1 mM MgCl_2_, 10 mM HEPES, 0.1% (wt/vol.) BSA, pH 7.4). Cells were then immersed in low glucose (LG, 2.8 mM) KRBB for a 1-hour pre-incubation at 37°C and 5% CO_2_ and the supernatant was discarded. Next, cells were immersed in 100 uL (96-well plate: INS-1 832/3 cells) or 500 uL (1.5 mL tubes: human islets) LG-KRBB for 1 hour, followed by high glucose (HG, 16.7 mM) KRBB for 1 hour at 37°C and 5% CO_2_. Supernatant from the LG-KRBB and HG-KRBB incubations was collected and stored at −30°C until analysis. To measure total insulin content, cells were immersed in acid ethanol (75% EtOH, 1.5% HCl: same volumes as above) overnight at 4°C, and supernatant was then neutralized with equal volume 1 M Tris-base (Fisher Bioreagents, #BP152–1; Pittsburgh, PA) and stored at −30°C until analysis. Insulin concentrations were measured by ELISA (INS-1 832/3: ALPCO Rodent Insulin Chemiluminescence ELISA, #80-INSMR-CH10, Salem, NH; human islets: ALPCO Insulin Chemiluminescence ELISA, #80-INSHU-CH10).

For SC-islets, 15 SC-islets were hand-picked into a 1.5 mL microcentrifuge tube (*n* = 4–6 technical replicates per biological replicate). SC-islets were washed with pre-warmed 0 mM glucose KRBB and then immersed in 500 uL LG-KRBB for a 1-hour pre-incubation at 37°C and 5% CO_2_. SC-islets were then sequentially immersed in 500 uL LG-KRBB for 1 hour, HG-KRBB for 1 hour, and KCl-KRBB (LG +30 mM KCl) for 1 hour at 37°C and 5% CO_2_. Supernatant from the LG-KRBB, HG-KRBB and KCl-KRBB incubations was collected and stored at −30°C until analysis. SC-islets were then lysed in acid ethanol as described above and samples were stored at −30°C until analysis. Human insulin concentrations were measured by ELISA (ALPCO, #80-INSHU-CH10). Since SC-islets have limited glucose responsiveness in our hands, we normalized insulin secretion to the vehicle control within each stimulus condition (LG, HG, KCl) and for total insulin content.

### 2.5. Perifusion assay for dynamic insulin secretion

We assessed dynamic insulin secretion by perifusion (Biorep Technologies, Miami Lakes, FL) in mouse islets exposed to DMSO or 1 nM DP (*n* = 4–5 mice per condition). For each mouse, 70 islets were hand-picked and loaded into Perspex microcolumns sandwiched between two layers of acrylamide-based microbeads (PERI-BEADS-20, Biorep Technologies). Islets were perfused for 48 minutes with 2.8 mM LG-KRBB at a rate of 100 μL/min to equilibrate the islets. Islets were subsequently perifused with the following protocol at a rate of 100 μL/min: 10 minutes with 2.8 mM LG-KRBB, 32 minutes with 16.7 mM HG-KRBB, 24 minutes with LG-KRBB, 20 minutes with 30 mM KCl-KRBB, and 14 minutes with LG-KRBB. Samples were collected every 2 minutes (200 μL per sample).

We also assessed dynamic insulin secretion in mouse islets exposed to DMSO, GLT, and GLT +1 nM DP (*n* = 4 mice per condition). For each mouse, 70 islets were hand-picked and loaded into Perspex microcolumns as described above. Islets were perfused for 40 minutes with 2.8 mM LG KRBB at a rate of 40 uL/min to equilibrate the islets. Islets were then perfused with the following protocol at a rate of 40 uL/min: 15 minutes with 2.8 mM LG KRBB, 35 minutes with 16.7 mM HG-KRBB, 20 minutes with LG-KRBB, 25 minutes with 30 mM KCl-KRBB, and 10 minutes with LG-KRBB. Samples were collected every 5 minutes (200 uL per sample).

Throughout the perifusion process, both islets and perifusion solutions were maintained at 37°C using the built-in temperature-controlled chamber, while the collection plate was cooled to 4°C with the tray cooling system. Samples were stored at −80°C until analysis. Insulin concentrations were measured by ELISA (ALPCO, #80-INSMR-CH10).

### 2.6. Gene expression analysis

Mouse islets were collected in Buffer RLT supplemented with 0.143 M 2-mercaptoethanol and stored at −80°C until use. RNA was isolated using the RNeasy Micro Kit (Qiagen, #74004). cDNA synthesis was accomplished using the iScript gDNA Clear cDNA Synthesis Kit (Bio-Rad, Cat#1725035). RT-qPCR was performed on experimental samples along with “no reverse transcriptase” (NRT) and “no template controls” (NTC) with SsoAdvanced Universal SYBR Green Supermix (Bio-Rad, #1725271) and run on a CFX384 with Bio-Rad CFX Maestro program. *Ppia* was used as the housekeeping gene and all data were analyzed using $2^{-\Delta \Delta \mathrm{Ct}}$ values. Primers are listed in Supplemental Table S2.

### 2.7. Statistical analysis

Statistical analyses and figures were generated using GraphPad Prism 10.2 software. Data were analyzed with one-way or two-way ANOVAs with Tukey post-hoc tests, as indicated in figure captions. Mouse insulin content values were analyzed with non-parametric one-way ANOVA. For all analyses, significance was determined as *p < .05, **p < .01 and ***p < .001. Data are presented as mean ± SEM. Technical versus biological replicate data are indicated in figure captions.

## 3. Results

### 3.1. The metabolic effects of systemic DP exposure in male mice are not driven by changes in islet morphology or endocrine cell composition

**Figure 1.**
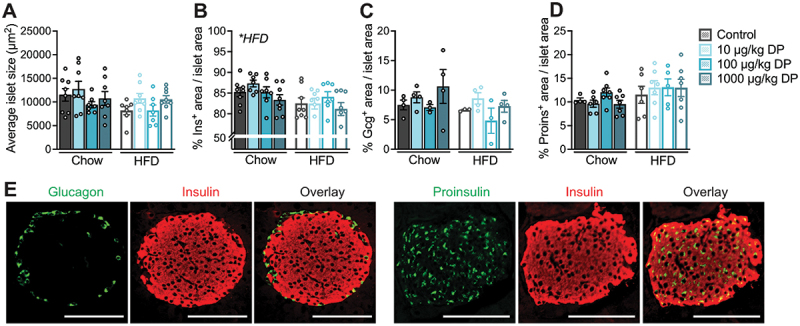
**The metabolic effects of *insystemicvivo* DP exposure in male mice are not driven by changes in islet morphology or endocrine cell composition.** Male mice were treated daily with vehicle or DP (10, 100, 1000 µg/kg per day) and fed chow or high fat diet (HFD) for 28 days; paraffin-embedded pancreas sections from these mice were analyzed by immunofluorescence staining to quantify islet morphology and endocrine cell composition. (a) Average islet size, (b) average % insulin (Ins)^+^ area, (c) average % glucagon (Gcg)^+^ area, and (d) average % proinsulin^+^ area per islet. (e) Representative images showing immunofluorescence staining of islets for insulin and glucagon, or insulin and proinsulin. Scale bars = 100 µm. All data are presented as mean ± SEM. Individual data points represent biological replicates (n = 3–8 biological replicates per condition). *p-value < .05 (two-way ANOVA with Tukey post-hoc).

We performed immunofluorescence staining on paraffin-embedded pancreas tissue sections from male mice exposed to vehicle or DP (10, 100, 1000 µg/kg) daily for 28 days and simultaneously fed either a chow or 60% HFD.^25^ Our goal was to determine if the glucose intolerance observed in DP-exposed HFD-fed mice relative to vehicle-exposed HFD-fed mice^25^ coincided with changes in islet morphology and/or pancreatic endocrine cell composition (Figure 1). We found a significant overall effect of HFD feeding to reduce the % insulin^+^ area per islet (Figure 1(b)), but no effect of DP exposure on any of the measured outcomes. Specifically, DP did not impact the average islet size (Figure 1(a)), % insulin^+^ area per islet (Figure 1(b)), % glucagon^+^ area per islet (Figure 1(c)) or % proinsulin^+^ area per islet (Figure 1(d)). Representative images of pancreatic islets showing insulin, glucagon, and proinsulin immunoreactivity are provided (Figure 1(e)). In summary, the glucose intolerance observed in DP-exposed HFD-fed mice^25^ is not explained by morphological changes to pancreatic islets. However, it is still plausible that DP caused glucose intolerance in part by disrupting insulin secretion, which cannot be assessed histologically.

### 3.2. DP exposure does not affect insulin secretion but reduces insulin content in INS-1 832/3 cells

**Figure 2.**
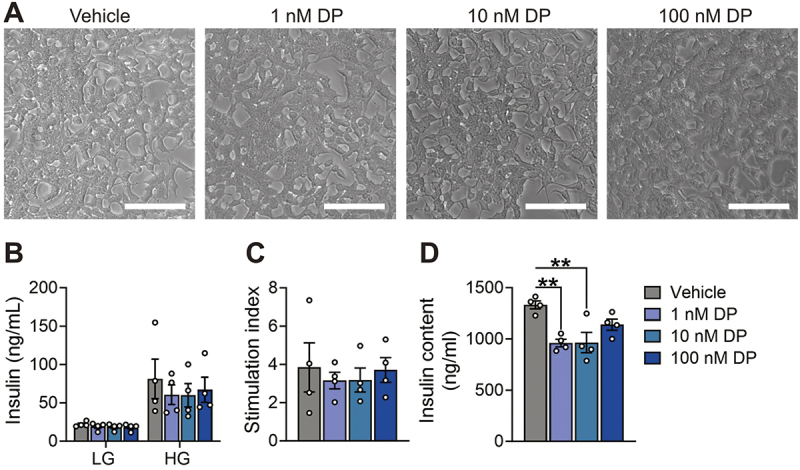
**DP exposure does not affect insulin secretion but reduces insulin content in INS-1 832/3 cells.** (a) Representative brightfield images of INS-1 832/3 cells treated with vehicle (DMSO) or DP (1, 10, 100 nM) for 48-hours *in* *vitro.* (b) Insulin secretion was quantified after a 1-hour static incubation in KRBB containing low glucose (LG; 2.8 mM) followed by 1-hour in high glucose (HG; 16.7 mM). (c) Stimulation index (HG:LG ratio). (d) Insulin content of lysed INS-1 832/3 cells. n = 6 technical replicates per biological replicate and n = 4 biological replicates per condition. Data represent mean ± SEM. Individual datapoints represent biological replicates. **p-value < .01 (one-way ANOVA with Tukey post-hoc; insulin content = non-parametric one-way ANOVA). Scale bars = 100 μm.

To investigate the impact of DP on β-cell function we started with the INS-1 832/3 rat β-cell line given their ease of use. These cells were exposed to DMSO (vehicle control) or DP for 48-hours *in vitro* and then static GSIS and total insulin content were assessed. DP did not affect overall cell morphology (Figure 2(a)), insulin secretion under LG or HG conditions (Figure 2(b)), or the HG:LG stimulation index (Figure 2(c)) in INS-1 832/3 cells. However, both 1 nM and 10 nM DP exposure significantly decreased insulin content from lysed cells (Figure 2(d)).

### 3.3. DP has no effect on insulin secretion or insulin content in human SC-islets

**Figure 3.**
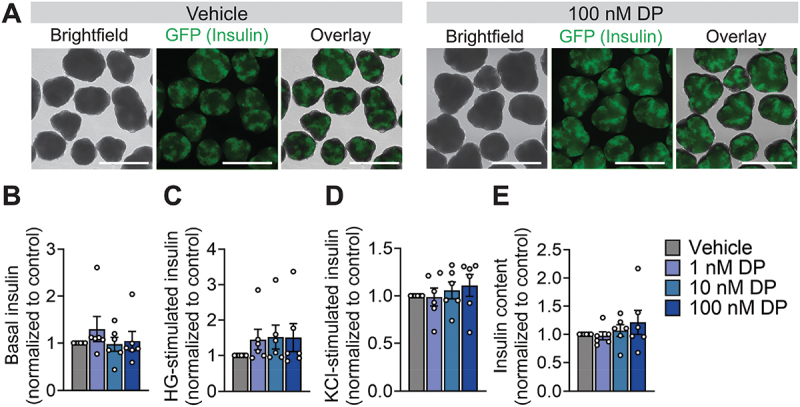
**DP has no effects on insulin secretion or insulin content in human SC-islets.** (a) Representative images showing SC-islet morphology following 48-hour exposure exposed to vehicle (DMSO) or DP (1, 10 nM). SC-islets were derived from INS-2A-EGFP stem cell line so GFP+ cells represent insulin+ cells. Insulin secretion in response to (b) low glucose (LG; 2.8 mM), (c) high glucose (HG; 16.7 mM), and (d) 30 mM KCl was assessed 48-hours post-exposure. (e) Total insulin content was measured in lysed cells. Data are normalized to the vehicle control condition for each endpoint (n = 6 biological replicates, i.e. differentiations per condition). Data represent mean ± SEM. Individual datapoints represent biological replicates. *p-value < .05 (two-way ANOVA with Tukey post-hoc). Scale bars = 750 µm.

Due to known differences between rodent and human responses to toxicant exposures,^32–34^ we next evaluated whether the effect of DP on insulin content would be reproducible in human β-cells. We first tested human SC-islets, which are a heterogeneous mixture of islet endocrine cells (e.g., β-cells, α-cells, δ-cells) and secrete human insulin, although with limited capacity to robustly secrete insulin in response to high glucose.^35^ Gross morphology of the differentiated INS-GFP^+^ cell clusters was not affected by DP exposure (representative images shown in Figure 3(a)). Pooling the results from 6 separate differentiations (i.e., biological replicates), we observed no overall effect of DP on insulin secretion after a 1-hour incubation in low glucose (Figure 3(b)), high glucose (Figure 3(c)), or 30 mM KCl (Figure 3(d)). There was also no effect of DP on total insulin content in SC-islets (Figure 3(e)).

### 3.4. Effects of DP on insulin secretion from human islets varies between donors

While SC-islets are a promising alternative to human donor islets, their lack of glucose responsiveness limits their applicability for assessing functional endpoints. Thus, we next analyzed the effects of DP on human islets isolated from five deceased organ donors. All donors were males between the age of 48–67 and with a body mass index (BMI) ranging from 24.9 to 37.4; three donors were non-diabetic (ND), and two donors were diagnosed with T2D (see Supplementary Table S1 for donor characteristics).

**Figure 4.**
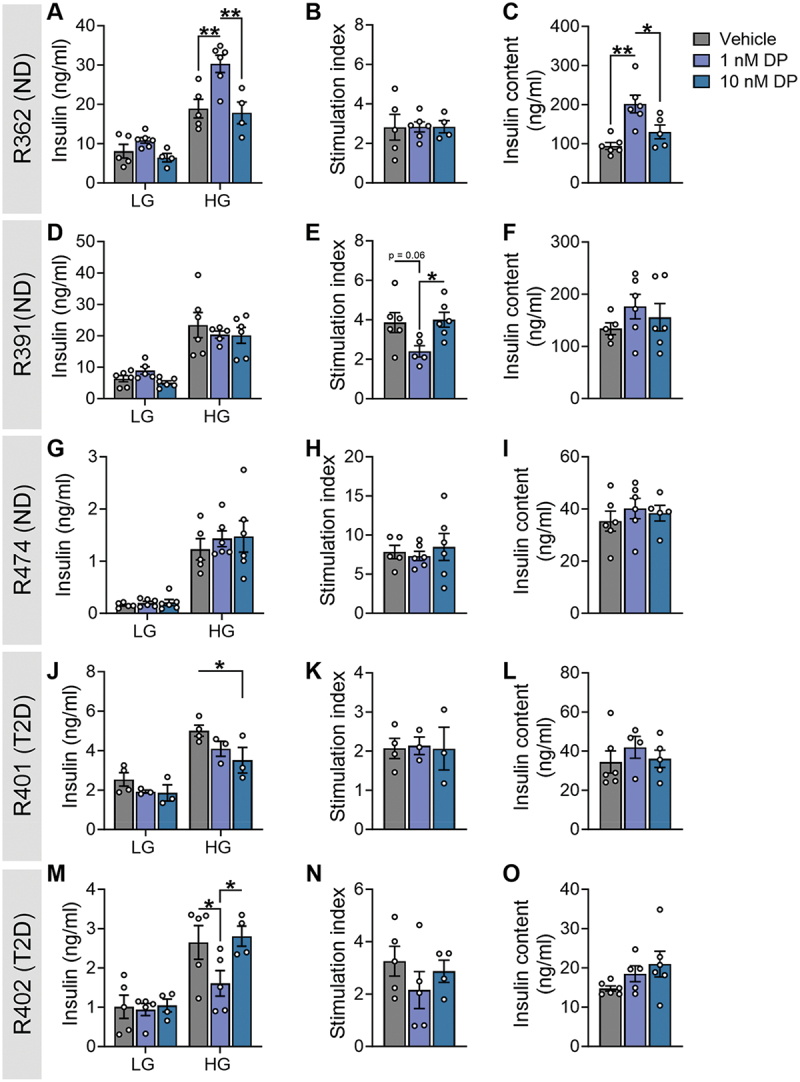
**Effects of DP on insulin secretion from human islets varies between donors.** Human islets from donors with no diabetes (ND; n = 3) or Type 2 diabetes (T2D; n = 2) were exposed to vehicle (DMSO) or DP (1, 10 nM) for 48-hours *ex vivo*. See Supplemental Table S1 for human donor characteristics. (a, d, g, j, m) Insulin secretion was quantified following a 1-hour static incubation in KRBB containing low glucose (LG; 2.8 mM) followed by high glucose (HG; 16.7 mM). (b, e, h, k, n) Stimulation index (HG:LG ratio). (c, f, i, l, o) Insulin content of islet lysates. All data represent the mean ± SEM. Individual datapoints represent technical replicates from a single donor. *p-value < .05, **p < .01 (GSIS: two-way ANOVA with Tukey post-hoc; stimulation index and insulin content: one-way ANOVA with Tukey post-hoc).

We observed notable biological variability in the effects of DP on human islets. DP-exposed islets from three of five donors exhibited altered insulin release under HG conditions, with ND donor R362 showing increased secretion in 1 nM DP conditions (Figure 4(a)) and T2D donors R401 and R402 showing decreased secretion in 10 nM or 1 nM DP conditions, respectively (Figure 4(j,m)). However, these changes in insulin concentration post-HG did not translate to significantly altered HG:LG stimulation index (Figure 4(b,k,n)). DP did not affect insulin secretion or the stimulation index in ND donors R391 (Figure 4(d,e)) and R474 (Figure 4(g,h)). DP exposure significantly increased insulin content in donor R362 (Figure 4(c)) but otherwise had no effect on insulin content in the other four donors (Figure 4(f,i,l,o)).

### 3.5. DP exposure alone does not alter insulin secretion or insulin content in mouse islets, but DP co-exposure with GLT stress decreases insulin content

To determine whether the reduced insulin content in DP-exposed INS1 832/3 cells (Figure 2) and/or altered insulin secretion in DP-exposed human islets (Figure 4) could be reproduced in another model, we next tested primary mouse islets. To further increase our confidence in the endpoint, we performed dynamic insulin secretion analysis via perifusion since this technique provides higher resolution data on 1^st^- and 2^nd^-phase GSIS, as well as KCl-induced insulin release.

**Figure 5.**
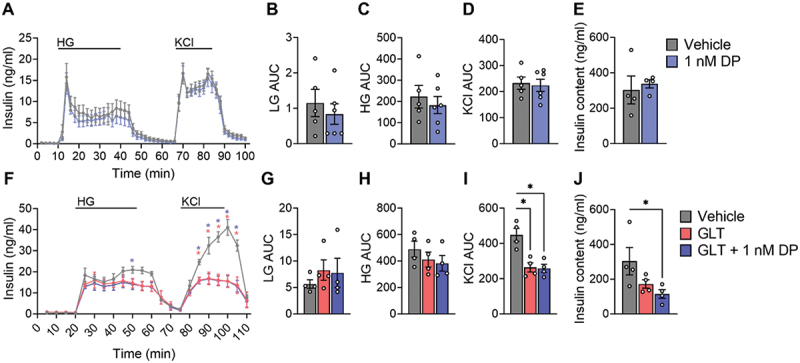
**DP alone does not alter insulin secretion or insulin content in mouse islets, but DP co-exposure with GLT reducesstress decreases insulin content.** (a-e) Mouse islets were exposed to vehicle (DMSO) or DP (1 nM) for 48-hours and dynamic insulin secretion was assessed in response to LG (2.8 mM), HG (16.7 mM), and KCl (30 mM). (f-j) Mouse islets were exposed to vehicle (DMSO), glucolipotoxic conditions (GLT: 28.7 mM glucose + 0.5 mM palmitate), or GLT conditions + 1 nM DP for 48-hours. (a, f) Dynamic insulin secretion curves from the perifusion assay and (b-d,g-i) area under the curve (AUC) for different periods of the perifusion. (e, j) Insulin content was measured in islets that underwent the same treatment conditions. All data represent mean ± SEM. Individual datapoints represent biological replicates (i.e. islets from different mice; n = 4-6 per group). *p < .05 (Perifusion curves: two-way ANOVA with Tukey post-hoc; stimulation index, AUCs: One-way ANOVA with Tukey post-hoc, Insulin content: non-parametric one-way ANOVA).

Consistent with the static GSIS results in INS-1 832/3 cells (Figure 2) and SC-islets (Figure 3), there was no effect of 1 nM DP exposure on either basal, glucose-stimulated, or KCl-induced insulin secretion in mouse islets (Figure 5(a-d)). Since it is challenging to recover islets from the perifusion chambers, we assessed the effects of DP on insulin content in a different subset of mouse islets that underwent the same exposure protocol (Figure 5(e)). In contrast to INS-1 832/3 cells (Figure 2(d)), DP did not affect insulin content in mouse islets (Figure 5(e)).

Since Peshdary, *et al*.^25^ reported that DP exposure amplified glucose intolerance in HFD-fed mice but not chow-fed mice, we next tested combined treatment of mouse islets with DP + GLT as a secondary metabolic stressor (Figure 5(f-j)). As expected, GLT conditions modestly impaired GSIS (Figure 5(f)) and robustly impaired KCl-induced insulin secretion (Figure 5(f,i)); the adverse effect of GLT on insulin secretion was not amplified by co-exposure with DP (Figure 5(f-i)). Interestingly, while there was no effect of DP alone (Figure 5(e)) or GLT alone (Figure 5(j)) on insulin content, the combined exposure to DP + GLT led to significantly reduced insulin content in mouse islets (Figure 5(j)).

### 3.6. DP amplifies the effect of GLT on *Slc30a8*

**Figure 6.**
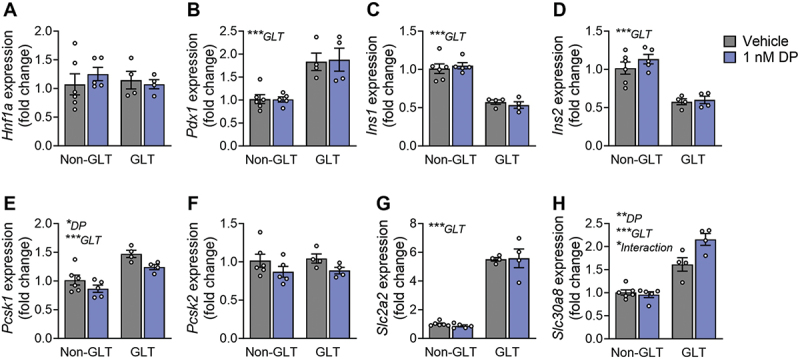
**DP amplifies the effect of GLT on *Slc30a8*.** Mouse islets were exposed to vehicle (DMSO), 1 nM DP alone, glucolipotoxic conditions alone (GLT: 28.7 mM glucose +0.5 mM palmitate), or combined GLT +1 nM DP for 48-hours. Gene expression for markers of β-cell maturity and function were analyzed by qPCR. All data represents mean ± SEM. Individual datapoints represent biological replicates (i.e. islets from different mice; n = 4-6 per group). *p < .05, **p < .01, ***p < .001 (two-way ANOVA with Tukey post-hoc).

Following exposure of mouse islets to DP ± GLT, we assessed expression of genes related to β-cell maturity and insulin secretion. *Hnf1a* was unchanged by GLT or DP exposure (Figure 6(a)), but *Pdx1* was significantly increased under GLT conditions (Figure 6(b)). Both *Ins1* and *Ins2* were significantly downregulated under GLT conditions but not affected by DP (Figure 6(c,d)). Interestingly, there was a significant overall effect of GLT to upregulate *Pcsk1* but a significant overall effect of DP to downregulate *Pcsk1* (Figure 6(e)); *Pcsk2* was not affected by either treatment condition (Figure 6(f)). Most notably, GLT conditions upregulated expression of both *Slc2a2* (Figure 6(g)) and *Slc30a8* (Figure 6(h)), but DP amplified the GLT-mediated effects on *Slc30a8* (Figure 6(h)).

## 5. Discussion

In the present study, we report that 28-day DP exposure *in vivo* did not affect mouse islet morphology. We also tested the effect of 48-hour DP exposure on diverse β-cell models *in vitro* and found no effect of DP on insulin secretion in INS-1 832/3 cells, SC-islets, or mouse islets (even in the presence of GLT stress), but modest effects of DP on GSIS in three of five human islet donors. DP significantly impaired intracellular insulin content in INS-1 832/3 cells but this finding was not replicated in other cell models. Interestingly, DP did reduce insulin content in mouse islets when cells were co-exposed to GLT stress conditions. These data support the hypothesis that DP interacts with other metabolic stressors and align with the original mouse study showing that DP amplifies glucose intolerance in HFD-fed but not chow-fed male mice.^25^ Finally, our study demonstrates the importance of model selection for toxicology studies due to species- and model-dependent differences in β-cell function in response to xenobiotic exposure.

Our study was prompted by interesting findings from Peshdary *et al*. showing that 28-days of oral low-dose DP exposure amplified glucose intolerance in HFD-fed mice and led to hyperinsulinemia in chow-fed mice.^25^ They also reported that *in vitro* DP exposure inhibited the insulin signaling pathway in rodent and human adipocytes.^25^ While impaired peripheral insulin sensitivity could explain DP-induced hyperinsulinemia and glucose intolerance, we speculated that DP may also dysregulate insulin secretion, either by disrupting β-cell function and/or β-cell mass. Importantly, hyperinsulinemia can be an early initiating event that subsequently drives the onset of peripheral insulin resistance.^36–39^ Although β-cell function was not assessed in the Peshdary study^25^, we were able to obtain fixed pancreas tissues from the vehicle- and DP-exposed mice to assess islet morphology. Our analysis indicated that DP did not alter islet size or endocrine cell composition of islets. We also quantified proinsulin immunoreactivity as a surrogate for β-cell function. In dysfunctional or stressed β-cells, insulin demand can exceed the ability of the endoplasmic reticulum to process newly translated proteins, leading to inadequately processed proinsulin being co-secreted alongside mature insulin.^40–43^ Proinsulin immunoreactivity was also not affected by DP exposure. However, these findings do not rule out potential effects of DP on insulin secretion.

To assess the effects of DP on β-cell function, we tested several complementary *in vitro* rodent and human model systems, each with advantages and limitations. The main differences between immortalized versus primary β-cells include: a) immortalized β-cells rapidly proliferate, whereas primary β-cells have minimal ability to replicate;^44,45^ b) immortalized β-cell lines lack the paracrine signaling from other islet endocrine cells; and c) immortalized β-cells are cultured in monolayer format, whereas primary islets are complex 3-dimensional spheroids. Mouse islets are a consistent, physiologically relevant, and “environmentally naïve” model for studying primary β-cells. However, there are important differences between human versus rodent β-cells that should be considered in toxicology testing.^32^^–46–48^

We are extremely fortunate to have access to high-quality primary human islets from deceased organ donors as a valuable resource.^49,50^ Human islets are more variable than mouse islets, a reflection of the inherent biological and environmental variability between organ donors,^51–53^ as well as differences that arise from islet isolation and processing parameters between donors.^50,54,55^ The ability to manufacture human β-like cells from stem cells provides a promising “environmentally naïve” human model for toxicity testing.^56^ However, the generation of fully mature SC-islets is still being optimized,^29–30–60^ and as of yet robust glucose responsiveness remains variable, which poses a limitation for studying toxicant effects on β-cell function. These diverse factors must be carefully considered when choosing an appropriate model(s) for toxicological assessments and comparing results across models.

Given the advantages and limitations of each cell model, it is important to interpret findings from each model cautiously. For example, while DP reproducibly reduced insulin content in immortalized β-cells, this finding was not replicated in human islets, human SC-islets, or mouse islets. Thus, the biological relevance of this finding is questionable and raises concerns about the applicability of toxicity data generated only using immortalized cell lines.^26,61^ The only model where we observed significant effects of DP on insulin secretion was in primary human islets. Although the diversity in biological responses between human donors poses a challenge in assessing toxicity testing results, it also reveals the spectrum of human β-cell vulnerability to environmental toxicants, which is contingent on multifaceted genetic and environmental variables. To limit biological variability, we restricted our selection criteria to male donors with a narrow age and BMI range (see Supplementary Table S1) and were fortunate to have access to three ND and two T2D donors for our study. Interestingly, of the three donors that showed altered GSIS following DP exposure, two were those with T2D. Given the small sample size, we are limited in our ability to fully investigate differences in the response of ND versus T2D islets to DP. However, we speculate that the complex environmental and genetic backgrounds of human islet donors may reveal interactions between DP and secondary T2D risk factors.

Peshdary *et al*. reported that DP caused hyperglycemia in HFD-fed mice, but not chow-fed mice.^25^ As such, we predicted that DP might elicit more robust effects on islets in the presence of a secondary metabolic stressor such as GLT conditions. Co-exposure of mouse islets to DP + GLT did not affect insulin secretion compared to either condition alone but did reveal an interactive effect between DP and GLT to reduce intracellular insulin content. Similarly, DP magnified the GLT-driven upregulation of *Slc30a8*, a zinc transporter (ZnT8) responsible for maintaining adequate zinc flux and subsequent insulin-vesicle packaging,^62^ in mouse islets. Overexpression of ZnT8 in mouse β-cells leads to severely impaired GSIS.^63^ Thus, the effect of DP to amplify GLT-mediated *Slc30a8* expression in mouse islets could have important implications for T2D pathogenesis.

One important limitation of our study is the focus solely on male β-cells, which overlooks the potential sex-specific effects of DP on β-cell function. Sex differences in toxicokinetics and adverse health outcomes following toxicant exposure are well established.^64^ Other flame retardants exert sex-specific effects on gene expression in brain and liver in mice^65^ and hormone concentrations in humans.^66^ Our study focused on male β-cell phenotypes as a starting point for investigating diabetogenic effects of DP to expand upon the study in male mice by Peshdary *et al*. and also to maintain consistency in biological sex between models since INS-1 832/3 and SC-islets both originate from males. However, future studies should investigate the effect of DP on female β-cells both *in vivo* and *in vitro.*

To our knowledge this is the first study to investigate the diabetogenic effects of Dechlorane Plus on pancreatic β-cells. Our study shows modest and variable effects of DP on insulin secretion in human islets but no notable detrimental effects on β-cell function in other models. DP also has model-dependent effects on intracellular insulin content. Collectively, these data highlight the importance and challenges of using different *in vitro* models for studying chemical toxicity.

## Abbreviations

DP: Dechlorane plus
GSIS: Glucose-stimulated insulin secretion
POPs: Persistent organic pollutants
SC-islets: Stem cell derived islet-like clusters
T2D: Type 2 diabetes
GLT: Glucolipotoxicity

## Supplementary Material

Graphical abstract file placeholder.docx

20240524_van Allen et al_Supplementary Data.docx
