# Supplementary material for: Characterizing the effects of Dechlorane Plus on β-cells: a comparative study across models and species
Source: Islets. 2024 Jun 4;16(1):2361996. doi: 10.1080/19382014.2024.2361996 (PMC11152096; doi:10.1080/19382014.2024.2361996)
Supplement: Graphical abstract file placeholder.docx [file KISL_A_2361996_SM0669.docx]

We are opting to not include a graphical abstract at this time.
