# Supplementary material for: Characterizing the effects of Dechlorane Plus on β-cells: a comparative study across models and species
Source: Islets. 2024 Jun 4;16(1):2361996. doi: 10.1080/19382014.2024.2361996 (PMC11152096; doi:10.1080/19382014.2024.2361996)
Supplement: 20240524_van Allen et al_Supplementary Data.docx [file KISL_A_2361996_SM0668.docx]

**Characterizing the effects of Dechlorane Plus on β-cells: a comparative study across models and species**

Kyle A van Allen^1^, Noa Gang^1^, Myriam P Hoyeck^1^, Ineli Perera^1^, Dahai Zhang^2^, Ella Atlas^3^, Francis C Lynn^2^, Jennifer E Bruin^1^*

^1^ Department of Biology & Institute of Biochemistry, Carleton University, Ottawa, Ontario, K1S 5B6 Canada.

^2^ Environmental Health Science and Research Bureau, Health Canada, Ottawa, ON

^3^ Diabetes Research Group, BC Children’s Hospital Research Institute, Vancouver, BC, Canada

* Address correspondence to:

Dr. Jennifer Bruin

1125 Colonel By Drive

Ottawa, ON K1S 5B6

T: 613-520-2600 x3656

[jenny.bruin@carleton.ca](mailto:jenny.bruin@carleton.ca)

Supplementary Table 1: Human organ donor characteristics from donor islets used in this study. Islets were isolated by Alberta Diabetes IsletCore (<https://www.epicore.ualberta.ca/IsletCore/>). BMI = Body Mass Index, T2D = Type 2 Diabetes, HbA1C = hemoglobin A1c.

| **Donor ID** | **BMI** | **Age** | **Biological Sex** | **T2D Status** | **HbA1C** |
| --- | --- | --- | --- | --- | --- |
| R362 | 29.4 | 54 | Male | No | N/A |
| R391 | 24.5 | 67 | Male | No | 4.9 |
| R401 | 37.4 | 51 | Male | Yes (10 years + Metformin) | 6.4 |
| R402 | 26.7 | 57 | Male | Yes (No treatment) | 6 |
| R467 | 24.9 | 48 | Male | No | 5.8 |

Supplementary Table 2: Primer list used for qPCR.

| **Target Name** | **Forward Primer Sequence** | **Reverse Primer Sequence** |
| --- | --- | --- |
| INS1 | TCA GAG ACC ATC AGC AAG CA | CTC CCA GAG GGC AAG CAG |
| INS2 | GCT TCT TCT ACA CAC CCA TGT | ACG ACT GAT CTA CAA TGC CAC |
| PCSK1 | GGT GGA AAG GTC GAG TCT AGC | TGC ACA CCA AAC GCA AAA GA |
| PCSK2 | TTT GGA GTC CGA AAG CTC CC | GGT GTA GGC TGC GTC TTC TT |
| HNF1A | CCG TGG TGG AGT CAC TTC TT | GAT GTT GTG CTG CTG CAA GT |
| PDX1 | GTA CGG GTC CTC TTG TTT TCC | GAT GAA ATC CAC CAA AGC TCA C |
| SLC2A2 | GCA ACT GGG TCT GCA ATT TT | CCA GCG AAG AGG AAG AAC AC |
| SLC30a8 | GGC TGA CAT TTG GGT GGT AT | TCA CAG GCA AGG TAC AGC AG |
